# Elephantiasis neuromatosa of the lower limb in a patient with neurofibromatosis type-1: A case report with imaging findings

**DOI:** 10.4103/1817-1745.66684

**Published:** 2010

**Authors:** Shahina Bano, Akhila Prasad, Sachchida Nand Yadav, Vikas Chaudhary, Namrata Sachdeva

**Affiliations:** Department of Radiodiagnosis, Dr. Ram Manohar Lohia Hospital and PGIMER, New Delhi - 110 001, India; 1 Department of Radiodiagnosis, Lady Hardinge Medical College and Smt. S.K. and Kalawati Hospitals, New Delhi - 110 001, India

**Keywords:** Computed tomography, conventional radiography, elephantiasis neuromatosa, magnetic resonance imaging, ultrasonography

## Abstract

Elephantiasis neuromatosa is the most impressive manifestation of neurofibromatosis type-1 (NF-1). We report a case of NF-1 who presented with elephantiasis neuromatosa of his right leg. Cross-sectional imaging not only assists in the correct diagnosis but also aids in imaging the vasculature of a plexiform neurofibroma, which is essential for proper surgical planning.

## Introduction

Neurofibromatosis type 1 (NF-1), previously known as Von Recklinghausen disease, is a phakomatosis or neurocutaneous syndrome with autosomal-dominant inheritance, primarily affecting the development and growth of nerve cell tissues, with a frequency of approximately 1 in 3,000 births.[1] Pathologically, neurofibromas in NF-1 can be divided into three types, the most common being localized neurofibroma, the least common being diffuse neurofibroma and the most characteristic lesion of the disease being plexiform neurofibroma.[2] Plexiform neurofibromatosis, pathognomonic of NF-1, exhibits a characteristic “bag of worms” appearance on gross examination and cross-sectional imaging because of the involvement of the long segment of a major nerve trunk and its branches.[3] This lesion, in its most extreme form, may involve an entire extremity, with gigantic hypertrophy of the skin, soft tissues and the underlying skeleton.[4] They may become very large and deformed, and is therefore named as “elephantiasis neuromatosa” by Virchow.[1]

## Case Report

**Figure 1:**
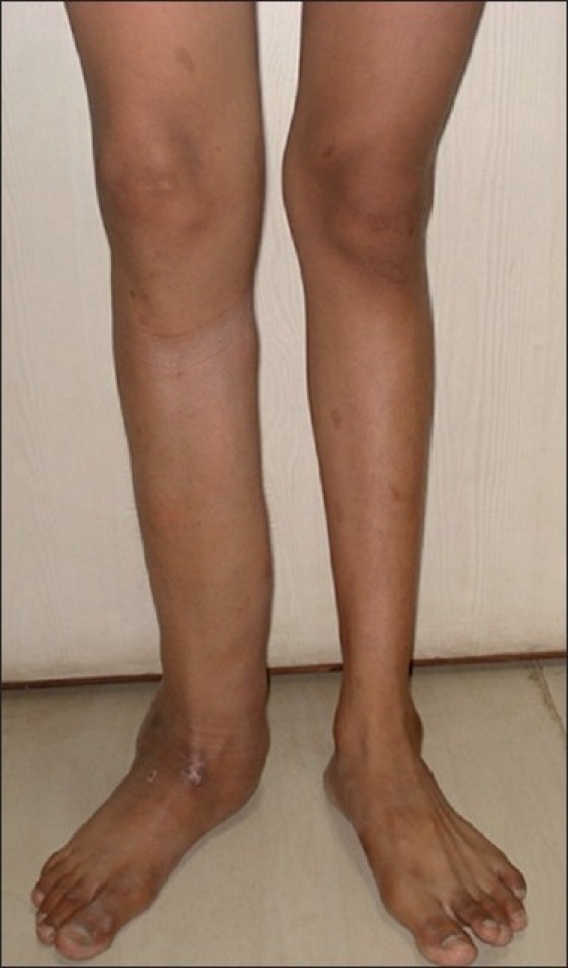
Photograph of bilateral lower extremities showing gross right limb enlargement, hypertrophy, disfigurement and skin ulceration

**Figure 2:**
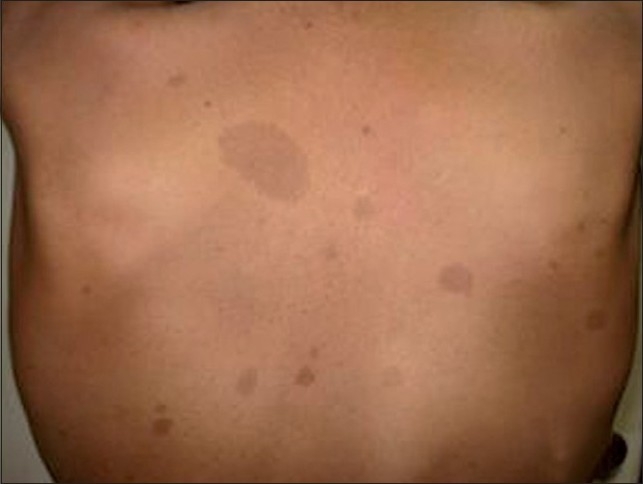
Photograph of the back of the trunk showing multiple “café au lait” spots of variable sizes

**Figure 3:**
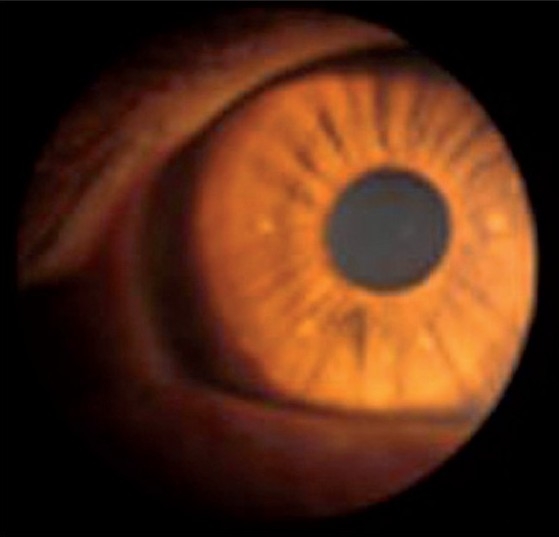
Slit lamp examination of the right eye showing multiple (five) pigmented iris hamartomas (Lisch nodules)

A 15-year-old male child with known NF-1 was referred to our radiology department for detailed assessment of a large disabling right leg plexiform neurofibroma that had recently undergone skin ulceration and had affected the patient’s gait. This slow-growing plexiform neurofibroma since childhood had resulted in gross limb enlargement and disfigurement [Figure 1], necessitating debulking surgery. Local examination revealed marked soft tissue hypertrophy of the right leg extending from the knee to the ankle. Clinically, the patient had no neurological or visual symptoms. However, physical examination revealed numerous “café au lait” spots (>5 mm in greatest diameter) randomly distributed all over the body [Figure 2], with axillary freckling. Slit lamp examination of both eyes revealed multiple pigmented iris hamartomas (Lisch nodules); five in the right eye [Figure 3] and three in the left eye. Blood and urine tests, including the vanillylmandelic acid test, were all within normal ranges. There was no family history of NF-1 in first-degree relatives.

**Figure 4:**
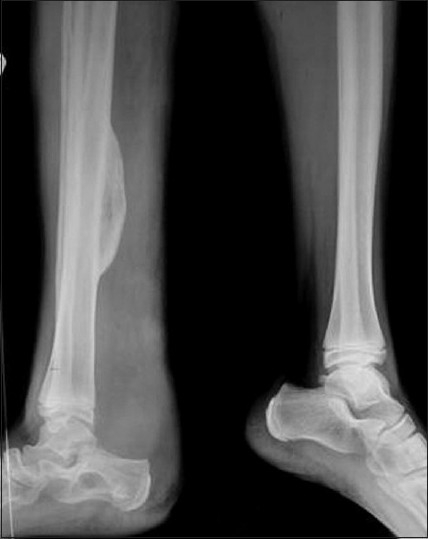
Conventional radiograph of the bilateral lower limbs shows disparity in limb length. The right limb appears relatively longer and shows soft tissue hypertrophy, periosteal and endosteal thickening (involving middle 1/3rd shaft of the tibia), scalloping of planter surface of right calcaneum and erosion of articular surfaces of the right talocalcaneal joint

**Figure 5:**
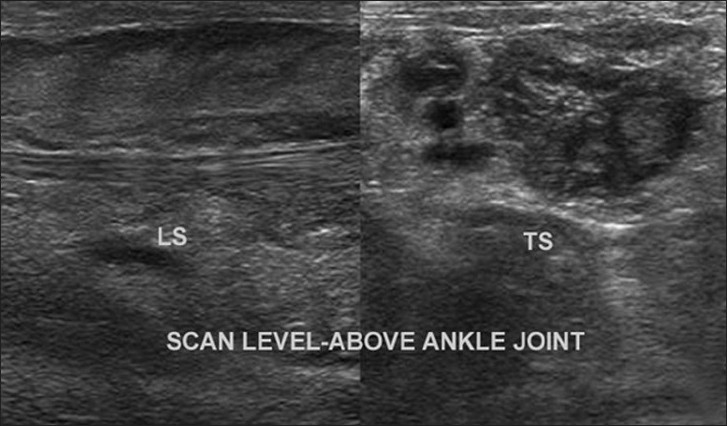
Gray scale ultrasonography of the right lower limb shows multilobulated tortuous entanglement of tumors, oriented along the long axis of the nerve on longitudinal section, and “target sign” on transverse scan, evident as echogenic center and hypoechoic periphery

**Figure 6:**
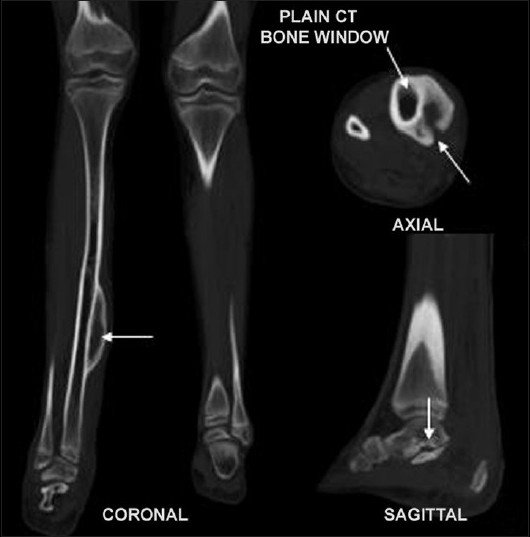
Plain computed tomography (bone window) demonstrates a large periosteal defect giving way to the infiltrating mass lesion, periosteal/ endosteal thickening (axial and coronal image-arrows) and cortical erosions at talocalcaneal joint (sagittal image-arrows)

**Figure 7:**
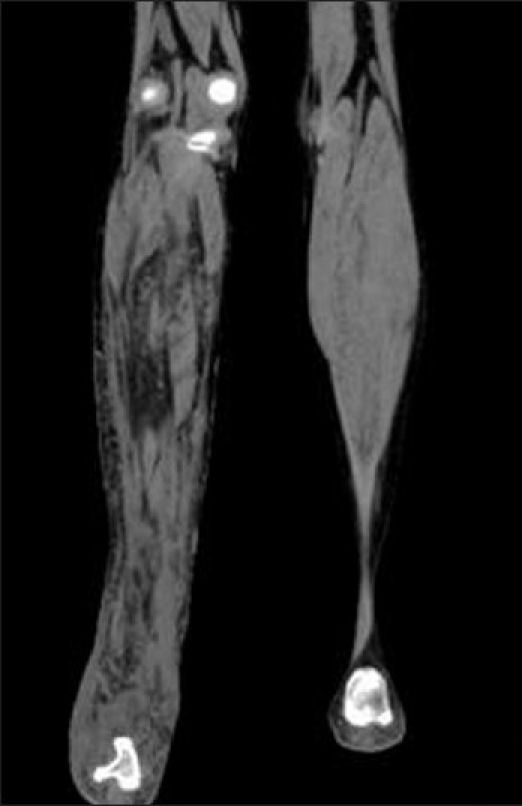
Plain computed tomography (soft tissue window) shows thick wavy cords of low attenuated masses traversing the soft tissue and giving a reticular-network appearance to the right leg

**Figure 8:**
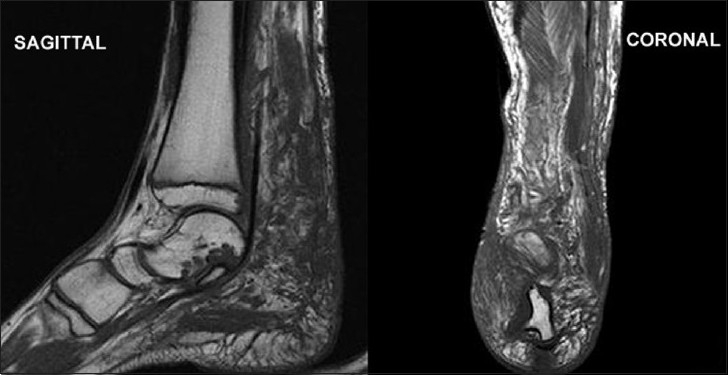
T1W sagittal and coronal sequences show marked atrophy and poor differentiation of individual muscles and variegated appearance of masses, ranging from nodular to thick irregular cords, few of them showing a branching pattern. The lesion shows signal intensity similar to as well as slightly higher than that of muscle

**Figure 9:**
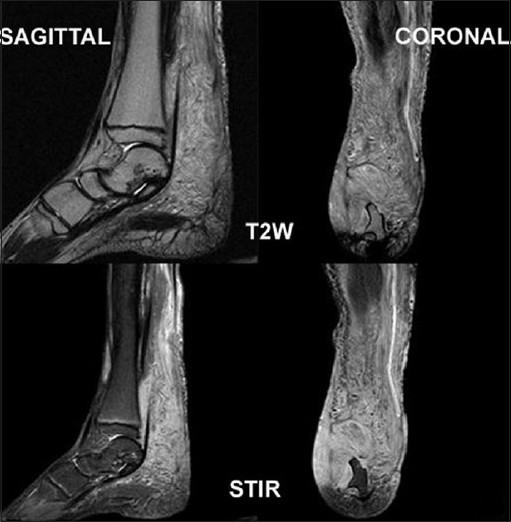
On T2W/STIR (sagittal and coronal) sequences, the lesion demonstrates a characteristic “bag of worms” appearance of plexiform neurofibromatosis

**Figure 10:**
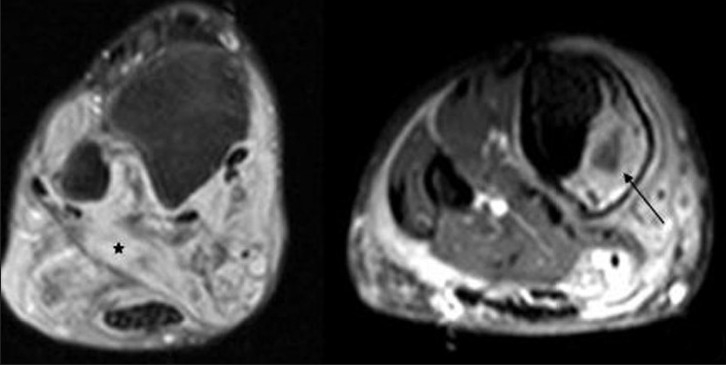
Postcontrast T1W fat saturation image reveals marked but slight inhomogenous enhancement of the soft tissue (asterisk) and subperiosteal component (arrow) of the mass. The subperiosteal component also shows a central area of cystic necrosis

**Figure 11:**
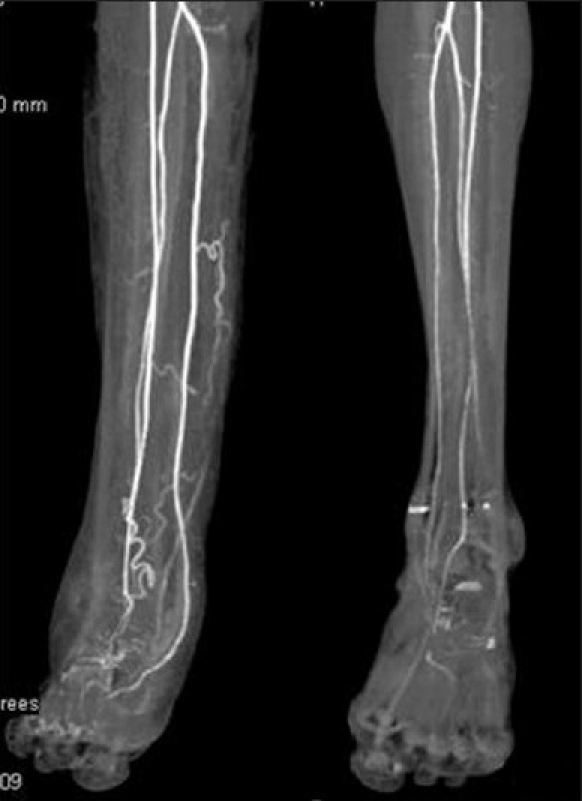
Dynamic 3D postcontrast computed tomography angiography (arterial phase) reveals hypertrophy of the right lower limb main arteries, which otherwise appear to be patent. Multiple tortuous collateral branches infiltrating soft tissues of the calf are also seen

Conventional radiograph showed relative lengthening and marked soft tissue hypertrophy of the right leg. Osseous abnormalities included thinning of bones, erosion of distal articular surfaces and periosteal dysplasia [Figure 4]. Gray scale evaluation revealed a diffuse infiltrative, interdigitating network of tumors oriented along the long axis of the nerve. The lesions were heterogeneous in nature and had characteristic “target sign” appearances. However, due to the diffuse infiltrative nature of the lesion, the entering/exiting nerve points could not be detected [Figure 5]. Plain computed tomography (CT) (bone and soft tissue window) confirmed the plain X-ray findings of periosteal dysplasia and cortical erosions. In addition, it also demonstrated an infiltrating network of low attenuated masses traversing the soft tissue and invading the periosteum through a large periosteal defect [Figures 6 and 7]. All magnetic resonance imaging (MRI) sequences demonstrated extensive, conglomerate soft tissue masses infiltrating the subcutaneous fat and multiple muscular compartments of the right leg, causing marked atrophy and poor differentiation of individual muscles. The masses had a variegated appearance, ranging from nodular to thick irregular cords, few showing a branching pattern. The lesion exhibited mixed signal intensity similar to, and slightly higher than, that of muscle on T1W sequence [Figure 8] and heterogeneously high signal intensity on T2W/STIR sequences giving a “bag of worms” appearance – the characteristic of plexiform neurofibromatosis [Figure 9]. Marked, but inhomogenous, enhancement of both the soft tissue and the subperiosteal component of the mass was evident on intravenous injection of gadolinium [Figure 10]. The dynamic contrast-enhanced 3D CT angiography revealed hypertrophy of the right lower limb vessels; all main arteries were enlarged but patent. There were multiple tortuous collateral branches that infiltrated the soft tissues of the calf [Figure 11]. The venous phase also revealed several enlarged draining veins (not shown).

**Figure 12:**
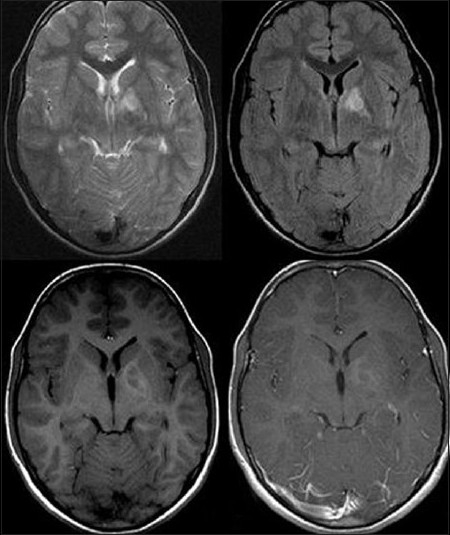
Screening magnetic resonance imaging of the brain shows a nonneoplastic hamartomatous lesion in the left basal ganglia

Screening MRI of the brain showed a well-defined lesion in the left basal ganglia, particularly involving the internal capsule, adjoining part of the globus pallidus and the thalamus. The lesion demonstrated mixed hypo- and hypersignal intensity on T1WI, high signal intensity on T2W/FLAIR sequences and no enhancement after contrast administration. The lesion typically did not exhibit mass effect, edema or contrast enhancement, suggesting non-neoplastic hamartoma [Figure 12].

The patient underwent partial tumor resection and cosmetic repair of the right leg. He was also advised follow-up MRI of the brain for hamartomatous lesion, which normally shows spontaneous regression with time.

**Figure 13:**
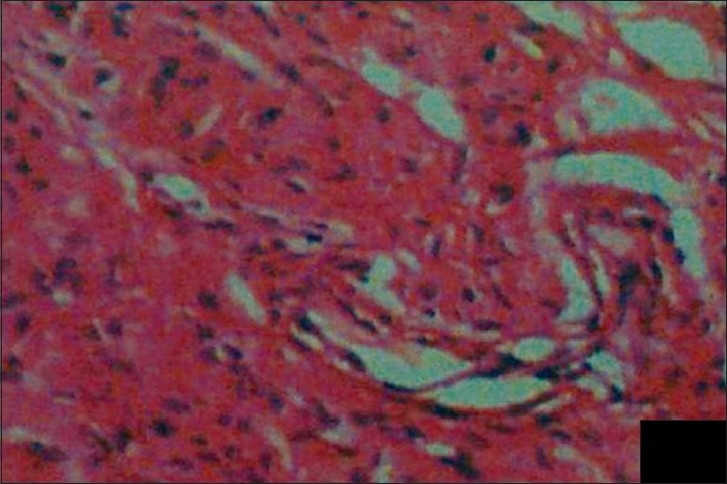
Histopathology of the resected mass reveals a large number of irregular infiltrative spindle cells, several nerve segments of varying length embedded in subcutaneous fat, many mast cells and myxoid stroma – finding consistent with diffuse plexiform neurofibromatosis

The histopathologic evaluation of the resected mass revealed a non-capsulated lesion comprising a large number of irregular infiltrative spindle cells and several nerve segments of varying length embedded in subcutaneous fat. The nerves were surrounded by a myxoid stroma that extended into the papillary dermis. Many mast cells were also seen within the diffuse component of the tumor. However, no mitosis was detected. The findings were suggestive of diffuse plexiform neurofibromatosis [Figure 13].

## Discussion

NF-1 is a hamartomatous disorder, with the genetic defect localized to the long arm of chromosome 17q11.2.[1] It is characterized by various skin lesions and peripheral or central nervous system neoplasm. The National Institutes of Health (NIH) in 1987 established diagnostic criteria of patients with NF-1. These well-recognized diagnostic criteria are neurofibromas (two or more simple, or one plexiform neurofibroma), café-au-lait spots (six or more, >5 mm in greatest diameter in children and >15 mm in adults), Lish hamartomas in iris (two or more), axillary or inguinal freckling, skeletal abnormalities (sphenoid dysplasias or cortical thinning, with or without pseudoarthrosis), optic glioma and first-degree relative with NF-1. Presence of two or more of these seven criteria establishes the diagnosis of NF-1. In our patient, five of the seven above-mentioned diagnostic features were present.[5]

Plexiform neurofibroma, found in up to 26% of patients with NF-1 is considered an uncommon skin tumor, usually presenting at birth or during the first several years of life. They are unencapsulated, poorly circumscribed tumors that diffusely infiltrate the nerve and the adjacent fat and muscle. As a result, neurofibromas are usually unresectable tumors, where tumor resection is impossible without sacrificing the nerve tissue. Fusiform enlargement of multiple nerve fascicles and branches is characteristic. Plexiform neurofibromas contain a mixture of Schwann cells, fibroblasts, reticulin and collagen fibers and a loose mucoid matrix interspersed between the axons of the parent nerve. They typically affect the trunk and extremities, but may also involve the head-neck and bladder. Associated bone dysplasia is often encountered secondary to chronic hyperemia or as part of the mesodermal dysplasia. Such tumors give rise to a variety of problems, including disfigurement and functional impairment.[6]

On ultrasound, these masses are usually hypoechoic, well defined and elliptical, oriented along the long axis of the nerve. Posterior acoustic enhancement is very common (70%). Color Doppler reveals variable vascular patterns, ranging from moderate, irregular central to predominantly peripheral vascularity. Some may not have any demonstrable vascularity.[7] MRI reveals large conglomerate masses consisting of innumerable neurofibromas, diffusely thickening the involved nerve and often extending into the nerve branches. This tumor has a locally aggressive behavior, but the infiltrative pattern is not indicative of malignancy and has no histologic evidence of anaplastic or mitotic features.[8] The typical pattern on MRI is relatively low signal intensity or signal intensity similar to that of muscle with T1-weighting and signal intensity greater than that of fat with T2-weighting. The hyperintense pattern on T2WI reflects the high water content of the myxoid matrix.[2] Three signs have been noted as diagnostic aids on MRI,[9] but they are also seen on ultrasound.[3] The “target sign” is characteristic of benign neurofibroma. The target appearance represents a geographic difference between the histologic zones of the neurofibroma. The high signal intensity seen in the peripheral zone is likely related to the high water content of the myxomatous tissue and the central low signal intensity is probably related to T2 shortening caused by the dense fibrocollageneous tissue. The fascicular sign refers to fascicular bundles in neurogenic tumors showing speckled appearance due to the presence of both high and low signal intensity. The split-fat sign is the presence of fine rind of fat at the periphery of the masses, and it represents the slow-growing nature of the tumor. The tumor usually exhibits avid contrast uptake but has a heterogeneous appearance on ultrasonography and MRI, owing to the presence of cysts, hemorrhage or necrosis.[3,9,10] CT of plexiform neurofibromas shows large multilobulated low-attenuation masses, usually within a major nerve distribution.[4] MR or CT angiography is mainly used to assess the vascular supply of the tumor and abnormal tumor vessels and to locate vessels suitable for preoperative intra-arterial embolization.[4,5] Large and diffuse masses may cause venous obstruction and hypertrophy of the feeding vessels. Postcontrast images may also show the extensive capillary pooling of contrast throughout the soft tissue mass corresponding to the plethora of abnormal vessels in “the hemangio-neurofibroma” and the large ectatic veins, which is a pathognomonic finding of a hypervascularised plexiform neurofibroma.[4]

The radiological modalities most often used in analyzing neurofibroma include CT and MRI. Ultrasound and color Doppler has a very limited role in the evaluation of a large mass, extending outside the range of the probe. Although CT is rarely helpful in making a specific diagnosis, it can provide a precise evaluation of the bone lesion and the extent of the soft tissue lesion; however, it is by far inferior to MRI in soft tissue contrast resolution and the visualization of tissue planes. Dynamic contrast-enhanced 3D MR or CT angiography represents a recent advance in imaging with rapid acquisition of high-quality angiographic images that permit a free choice of imaging planes and phases delineating the arterial and venous supplies, visualization of the abnormal changes of the vasculature in the affected limb, important landmarks in surgical planning. It should be emphasized that MRI and MR angiography may assist not only in the correct diagnosis of neurofibroma but also in imaging the vasculature of a plexiform neurofibroma, which is essential for proper surgical planning.

Treatment of diffuse and progressive plexiform neurofibroma is primarily surgery. However, complete resection of the tumor is not possible because of marked entanglement of the tumor with the nerves. Improved understanding of the molecular and cellular biology of the cells involved in the formation and growth of neurofibromas has lead to development of other forms of treatments, including drug therapies, whose role is yet to be defined.[10]

The common differential diagnosis in this case is other soft tissue tumors causing elephantiasis, such as filariasis, macrodystrophia lipomatosa, lymphangiomatosis, vascular malformation such as hemangioma and massive subperiosteal hematoma.

## Conclusion

There are many imaging modalities available today to study the peripheral nerves. It should be emphasized that MRI and angiography (MR or CT angiography) may assist not only in the correct diagnosis of neurofibroma but also in imaging the vasculature of a plexiform neurofibroma, which is essential for proper surgical planning.
